# Variable calling of m^6^A and associated features in databases: a guide for end-users

**DOI:** 10.1093/bib/bbae434

**Published:** 2024-09-11

**Authors:** Renhua Song, Gavin J Sutton, Fuyi Li, Qian Liu, Justin J-L Wong

**Affiliations:** Epigenetics and RNA Biology Laboratory, School of Medical Sciences, The University of Sydney, Camperdown, NSW 2050, Australia; Faculty of Medicine and Health, The University of Sydney, Camperdown, NSW 2050, Australia; Epigenetics and RNA Biology Laboratory, School of Medical Sciences, The University of Sydney, Camperdown, NSW 2050, Australia; Faculty of Medicine and Health, The University of Sydney, Camperdown, NSW 2050, Australia; College of Information Engineering, Northwest A&F University, Yangling 712100, Shaanxi, China; South Australian immunoGENomics Cancer Institute (SAiGENCI), The University of Adelaide, Adelaide, South Australia 5005, Australia; Nevada Institute of Personalized Medicine, University of Nevada, Las Vegas, Maryland Pkwy, NV 89154, United States; School of Life Sciences, College of Sciences, University of Nevada, Las Vegas, Maryland Pkwy, NV 89154, United States; Epigenetics and RNA Biology Laboratory, School of Medical Sciences, The University of Sydney, Camperdown, NSW 2050, Australia; Faculty of Medicine and Health, The University of Sydney, Camperdown, NSW 2050, Australia

**Keywords:** m^6^A, RNA modification, bioinformatics databases, SNPs, iM6A

## Abstract

N6-methyladenosine (m$^{6}$A) is a widely-studied methylation to messenger RNAs, which has been linked to diverse cellular processes and human diseases. Numerous databases that collate m$^{6}$A profiles of distinct cell types have been created to facilitate quick and easy mining of m$^{6}$A signatures associated with cell-specific phenotypes. However, these databases contain inherent complexities that have not been explicitly reported, which may lead to inaccurate identification and interpretation of m$^{6}$A-associated biology by end-users who are unaware of them. Here, we review various m$^{6}$A-related databases, and highlight several critical matters. In particular, differences in peak-calling pipelines across databases drive substantial variability in both peak number and coordinates with only moderate reproducibility, and the inclusion of peak calls from early m$^{6}$A sequencing protocols may lead to the reporting of false positives or negatives. The awareness of these matters will help end-users avoid the inclusion of potentially unreliable data in their studies and better utilize m$^{6}$A databases to derive biologically meaningful results.

## Introduction

N6-methyladenosine (m$^{6}$A) is the modification to adenosine (A) in RNAs via the addition of a methyl group at the nitrogen-6 position. It is the most prevalent, abundant, and conserved modification to messenger RNA (mRNA) molecules [1–5], but has also been found in other RNA species including long non-coding RNAs and circular RNAs [6–12]. In mature mRNAs, m$^{6}$A is typically enriched in the 3$^{\prime }$ UTRs and near the stop codons [13] and within a consensus motif, DRACH (D=A, G or U; R=G or A; H=A, C or U) [2, 13, 14]. m$^{6}$A has been implicated in myriad cellular processes including mRNA transcription, stability, splicing, export, and translation [15–18]. Not surprisingly, aberrant levels of m$^{6}$A have been associated with the development of diverse human diseases [19–26].

**Figure 1:**
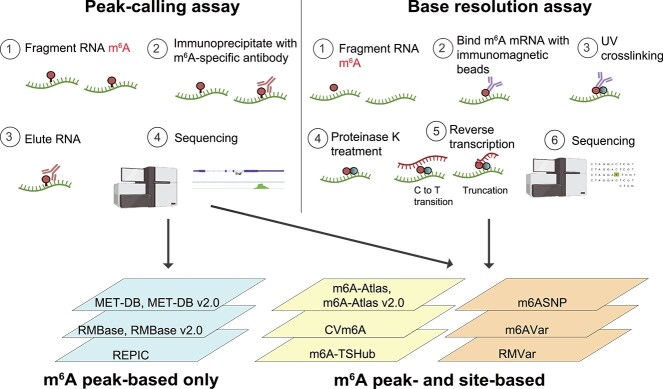
Experimental methods used to profile m$^{6}$A and databases that catalogue the resulting data; databases incorporate m$^{6}$A peaks from publicly available m$^{6}$A-seq and RIP-seq data sets or m$^{6}$A sites from publicly available miCLIP and miCLIP2 data sets. Parts of the figure were generated using Bio-Render.

To understand the roles of m$^{6}$A in human diseases and biological processes, various techniques have been developed to profile m$^{6}$A. Two widely used techniques are specific antibody-based high-throughput m$^{6}$A sequencing and crossing linking-assisted m$^{6}$A sequencing (Fig. 1). Antibody-based RNA immunoprecipitation (IP) uses high-throughput sequencing with peak-calling [2, 13, 27]. m$^{6}$A- and meRIP-seq [2, 13, 27, 28] are the earliest and most commonly used methods under this class of techniques. They profile m$^{6}$A methylated regions as peaks in transcript coverage from immunoprecipitated RNA relative to input RNA. The early version of this protocol [29] typically requires 300 $\mu $g of total RNA, which limits its application to cell lines. Subsequently, a refined protocol was developed by reducing the amount of total RNA to as low as 0.5 $\mu $g and has been applied to low-input RNA samples, including those obtained from primary human tumors [27]. Both early and refined methods map m$^{6}$A enrichment within 200-nt peak regions, and cannot precisely identify methylated adenosine at a single-base resolution [13]. To overcome this issue, novel techniques, such as crossing linking-assisted m$^{6}$A sequencing, were developed to accurately detect m$^{6}$A at single-nucleotide resolution. These methods include miCLIP [30, 31], PA-m$^{6}$A-seq [32], m$^{6}$A-REF-seq [33], MAZTER-seq [34], DART-seq [35], miCLIP2 [36], and scDART-seq [37]. Among them, miCLIP/m$^{6}$A-CLIP is well known for single-nucleotide detection of m$^{6}$A methylation sites by crosslinking at the antibody binding site to induce mutations of methylated bases during reverse transcription. However, these techniques may give rise to a considerable level of background signal due to the limited specificity of antibodies.

Nevertheless, consequent to implementing these m$^{6}$A detection methods, a large amount of m$^{6}$A modification data is now publicly available. To collate these data, several bioinformatics web servers and databases have been designed to collect, organize, integrate, and annotate m$^{6}$A data sets (Fig. 1) [38–50]. These databases can be categorized into three main groups. The first group of databases provides the status of RNA modifications based only on enriched peaks over input control obtained from immunoprecipitation sequencing experiments using antibodies against specific RNA modification marks. MeT-DB is the first epitranscriptome database that compiled the m$^{6}$A profiles for seven species in 26 different studies with a total of 74 meRIP-seq samples from 22 different experimental conditions [42, 43]. It contains additional functional data such as microRNA (miRNA) target sites, single nucleotide polymorphisms (SNPs), binding sites of splicing factors and RNA-binding proteins (RBPs), and information related to cancer genes. RMBase [40, 41] is another comprehensive epitranscriptome sequencing database that covers more than 100 types of RNA modifications in 13 species from 47 independent studies. These RNA modifications include pseudouridine ($\psi $) modifications, 5-methylcytosines (m$^{5}$C), m$^{6}$A, and 2$^{\prime }$-O-methylations (2$^{\prime }$-O-Me). RMBase provides novel web-based visualization tools for metagene profiles, logos of motifs associated with specific modifications, and various types of genomic features. In addition, it integrates epitranscriptome sequencing data to explore post-transcriptional modifications of RNAs and their relationships with miRNA binding events, RBP binding sites, disease-related SNPs, and genome-wide association study (GWAS) data. Recently, REPIC was released to record $\sim $10 million peaks from 672 samples of 49 independent studies, covering 61 cell lines or tissues in 11 organisms [44]. It also integrates 1418 histone ChIP-seq and 118 DNase-seq data to present a comprehensive atlas of m$^{6}$A methylation sites, histone modification sites, and chromatin accessibility regions. Accordingly, it allows users to explore cell/tissue-specific m$^{6}$A modifications and to investigate potential interactions between m$^{6}$A modifications and histone marks or chromatin accessibility.

The second group of databases collects m$^{6}$A both with peak-calling and at a single-base resolution from crossing linking-assisted m$^{6}$A sequencing. For instance, CVm6A compiles both m$^{6}$A-seq and miCLIP-seq/PA-m$^{6}$A-seq datasets in 23 human and eight mouse cell lines, and provides a visualization interface for searching and comparing the m$^{6}$A patterns in different cell lines [45]. Another database, m6A-Atlas, assembles over 400 000 high-confidence m$^{6}$A sites, and investigates cross-species conservation of m$^{6}$A sites among several species: seven vertebrate species (including human, mouse, and chimpanzee), 10 virus species (including HIV, KSHV, and DENV), and their host cells [46]. More recently, an updated version of m$^{6}$A-Atlas (v2.0) was generated to include nearly 800 000 reliable m$^{6}$A sites from 13 high-resolution methods and over 16 million m$^{6}$A peaks from meRIP-seq experiments [50].

Based on high confidence m$^{6}$A sites and m$^{6}$A peaks from datasets, some databases have included genetic variants that affect m$^{6}$A modification sites. Thus, the third group of databases provides information concerning the effects of genetic variants on m$^{6}$A to understand how they alter RNA methylation status to impact biological functions. For example, m6ASNP [47], m6AVar [48], and RMVar [51] adopted a random forest model to predict whether the methylation status of a m$^{6}$A site is altered by the disease-associated genetic variants within and flanking m$^{6}$A sites in human and mouse. m6AVar [48] and RMVar [51] collect a large number of m$^{6}$A-associated variants derived from millions of germline and somatic variants from miCLIP, PA-m$^{6}$A-seq, and meRIP-Seq experiments. The m$^{6}$A-associated variants are determined based on whether they localize to RBP-binding regions, splicing, and miRNA target sites. RMVar also explores the underlying relationship between the m$^{6}$A machinery and diseases by integrating the disease-association data from GWAS [52] and ClinVar [53] databases. m6A-TSHub presents a comprehensive platform for context-specific m$^{6}$A methylation and m$^{6}$A-affecting mutations in 23 human tissues in four key components (m6A-TSDB, m6A-TSFinder, m6A-TSVar, and m6A-CAVar) [49]. Inspired by m6AVar, m6A-Atlas [46] and m6A-Atlas v2.0 [50] inferred whether disease-associated genetic mutations could directly destroy the m$^{6}$A forming motif DRACH. A detailed summary of these databases is documented in Table 1.

**Table 1:** A detailed summary of m$^{6}$A databases

Databases	Functions	Modifications	Samples
MeT-DB [42] Website not available	binding sites of miRNA, splicing factor (SF), RBPs	m$^{6}$A	74 meRIP-seq from 22 different conditions
MeT-DB v2.0 [43] www.xjtlu.edu.cn/metdb2	miRNA target sites, SF binding sites,RBPs,Cancer related genes	m$^{6}$A	185 samples for seven species from 26 independent studies
RMBase [40] Website not available	protein genes, miRNA target sites, disease-related SNPs, regulatory ncRNAs	$\psi $ , m$^{5}$C, m$^{6}$A and 2$^{\prime }$-O-Me	18 independent studies
RMBase v2.0 [41] https://rna.sysu.edu.cn/rmbase	miRNA targets, RBP binding sites, SNVs and GWAS data	m$^{6}$A, m$^{1}$A, $\psi $, m$^{5}$C, 2$^{\prime }$-O-Me	47 studies among 13 species
CVm6A [45] http://gb.whu.edu.cn:8080/CVm6A	regulation of cell-dependent m$^{6}$A modification in disease and development	m$^{6}$A	23 human and eight mouse cell lines
REPIC [44] https://repicmod.uchicago.edu/repic	cell- or tissue-specific m$^{6}$A modifications, histone marks or chromatin accessibility	m$^{6}$A	672 samples of 49 studies, 61 cell lines in 11 organisms
m6ASNP [47] http://m6asnp.renlab.org	if methylation status of an m$^{6}$A site is altered by variants around the site	m$^{6}$A	59 234 human m$^{6}$A sites from [31] and [30]
m6AVar [48] http://m6avar.renlab.org	RBPs, miRNA-targets and splicing sites associated with variants, GWAS and ClinVar	m$^{6}$A	2 PA-m$^{6}$A-seq,7 miCLIP, 244 meRIP-Seq and a prediction based on Random Forest algorithm
RMVar[51] http://rmvar.renlab.org	RBPs, miRNA targets, splicing events, circRNAs and isease-related information from ClinVar and GWAS	m$^{6}$A/m, m$^{1}$A, m$^{5}$C, $\psi $, m$^{5}$U, 2$^{\prime }$-O-Me, A-to-I and m$^{7}$G	150 independent studies in human and mouse
m6A-Atlas [46] http://180.208.58.19/m6A-Atlas	RBPs, miRNA targets and splicing sites	m$^{6}$A	67 datasets from seven base-resolution methods and 1363 m$^{6}$A-seq
m6A-Atlas v2.0 [54] http://rnamd.org/m6a/	RBPs, miRNA targets, SNPs, and splicing sites	m$^{6}$A	2712 MeRIP-seq and 109 base-resolution samples
m6A-TSHub [49] http://180.208.58.19/tshub	RBPs, miRNA targets, and splicing sites, along with their known disease and phenotype linkage integrated from GWAS and ClinVar	m$^{6}$A	23 healthy human tissues and 25 tumour conditions

All these m$^{6}$A-related databases have been created with the aim of providing useful information to study m$^{6}$A-associated functions, but the lack of understanding of what these data present may lead to misinterpretation of m$^{6}$A. First, databases include meRIP-seq experiments that lacked reproducibility even when data were generated in the same cell types [55]. meRIP-seq experiments are also unable to determine the stoichiometry of m$^{6}$A. The sensitivity of m$^{6}$A calling is highly variable between different cells and tissues, making the interpretation of m$^{6}$A challenging. In addition, databases include datasets generated using both early and refined m$^{6}$A profiling methods that vary in sensitivity and specificity. Second, cell/tissue-specific m$^{6}$A profiles are not available in several of these databases, hindering m$^{6}$A investigations for specific cells or tissues. Indeed, certain m$^{6}$A modifications are tissue-dependent and are dynamically altered in response to different stimuli [56–60]. Third, reliable m$^{6}$A sites that can be detected using improved technologies including GLORI [61] and eTAM-seq [62] on new datasets are not yet incorporated into databases. Base-resolution datasets, often generated using technologies like miCLIP [30, 31], remain the minority in databases due to the comparative difficulty to generate them and consequent rarity in the literature (e.g. only 109/2821 samples (3.6%) in m6A-Atlas v2.0). Fourth, main analysis methods, such as MACS2 and exomePeak used to analyse m$^{6}$A datasets in databases, do not model the variation of methylation within transcripts and across biological replicates. Fifth, databases detailing m$^{6}$A-related SNPs have included genetic variants in sequences that flank the DRACH motifs, but these variants are not known to affect m$^{6}$A modification. Sixth, databases have included m$^{6}$A-related SNPs that contribute to non-synonymous variants. Therefore, it is unclear whether disease pathogenicity is consequent to altered amino acid sequence or m$^{6}$A function(s).

In this study, we explore currently available m$^{6}$A databases and describe their characteristics and limitations. We start by determining the number of m6A peaks and sites in specific cell lines reported in databases, highlighting a huge variability in the reported numbers. Next, as a significant portion of datasets compiled in databases was generated using the early m$^{6}$A-RIP-seq methods [2, 28, 29], we compared an example of the dataset obtained with the early method to the one obtained using the refined m$^{6}$A-RIP-seq protocol [27] in the same cell-line. We pinpoint potential errors in the early m$^{6}$A profiling method, which implies the need for additional filtering to minimize erroneous calling of m$^{6}$A. We describe the challenges of linking m$^{6}$A-associated SNPs to disease pathogenicity when these SNPs also constitute non-synonymous variants. Finally, we propose future directions towards generating better m$^{6}$A-related databases from an end-user perspective.

## Materials and methods

### Evaluation of peak variability within accessions across databases

To explore how peak calls varied in response to differences in the preprocessing pipelines of databases, we compared database peak coordinates derived from two public accessions: NOMO-1 m6A-seq from Su et al. 2018 [63] (GSE87190, with input in GSM2324291, and immunoprecipitation in GSM2324292); and H1299 meRIP-seq from Lin et al. 2016 [64] (GSE76367, with input in GSM1982262, immunoprecipitation in GSM1982263). We note these two cell-types were chosen as each has only a single m6A-seq / meRIP-seq sequencing run in the literature; this controls for one source of variability across databases, which is that when a cell-type has multiple sequencing runs, some databases call peaks separately per run (e.g. REPIC), while others derive a combined peak list from processing all runs together and thus uses more information (e.g. m6A-TSHub). For m6A-Atlas and m6A-TSHub, coordinates were converted from hg19 to hg38 using rTrackLayer [65]. For database pipelines using MACS2, intergenic peaks were excluded from analyses. Peaks coordinates were standardized to a width of 125bp centred around the midpoint, and intersected using intersectBed from the BedTools suite [66], requiring a minimum overlap of 1bp.

### Comparison of m$^{6}$A peaks detected in the human A549 cell line using early and refined m$^{6}$A-seq

To demonstrate variable calling of m$^{6}$A generated by widely used m$^{6}$A-seq protocols in existing databases, we compared the m$^{6}$A peaks identified using the early m$^{6}$A-seq protocol [67] and the refined RIP-seq protocol [27] in human A549 cell line. Data generated using either methods are publicly available for this cell line, and have been incorporated into m$^{6}$A-related databases. To call m$^{6}$A peaks, adaptor sequences, and low-quality reads were first trimmed from raw reads using Trimmomatic v0.38 [68] under default settings. FastQC v0.11.8 [69] was used to assess read quality, and STAR v2.5.2a [70] aligner was used to align clean reads to the human reference genome hg38 (ENSEMBL version 86). Samtools v1.6 [71] was used to select uniquely mapped reads to minimize the rate of false positives. MACS2 v2.1.0 [72] was used to call peaks enriched in immunoprecipitated over corresponding input samples. Peaks with enrichment scores >4 were selected for further analysis.

### Validation of A549 cell line-associated peaks

To identify potential false positive m$^{6}$A, a total of 10 704 high confidence m$^{6}$A sites in the human A549 cell line quantified by the m$^{6}$A-CLIP were downloaded from the m6A-Atlas database [46]. Bedtools was used to validate the m$^{6}$A peaks identified using the early m$^{6}$A-seq protocol and refined RIP-seq protocol by intersecting them with the existing high confidence m$^{6}$A sites [66]. A pie chart was used to visualize the percentage of verified m$^{6}$A sites in each method.

### Calculation of m$^{6}$A scores by an intelligent m6A (iM6A) model for m$^{6}$A sites in A549 cell lines

The intelligent m6A (iM6A) model was further used to show the potential erroneous m$^{6}$A sites generated by the early m$^{6}$A-seq protocol [67] and the refined RIP-seq protocol. To do this, the narrow peak output of MACS2 was expanded to +/- 250bp from its peak position. A 501bp sequence is used as input of iM6A (a deep learning model) to calculate the probability of the site being a m$^{6}$A site, consistent with the original article describing this model [73]. Each position in the peak sequence was assigned a probability ranging from 0 to 1: the larger the score, the higher the probability of a site being m$^{6}$A-methylated. The maximum probability of the sequence is used as m$^{6}$A probability of the called peaks. The line and box plots were used to visualize the difference in the m$^{6}$A probability between the early m$^{6}$A-seq protocol and the refined RIP-seq protocol.

## Results and discussion

### Existing databases are affected by highly variable calling of m$^{6}$A peaks

Although existing m$^{6}$A databases have been useful in providing a wealth of m$^{6}$A profiles of different cell-types and tissues, there are inherent complexities that may impact the meaningful derivation of data. Non-specialist end-users usually pay little attention to these complexities and neither do they realize that these complexities affect the reliability of m$^{6}$A profiles reported in these databases. The awareness of these complexities will help end-users to be more cautious when extracting data directly from these databases and understand how to make the most of these invaluable resources.

In most databases, m$^{6}$A enrichment in RNA transcripts is determined by peak calling which usually involves five steps. (1) QC: check reads’ quality; (2) trimming: remove adapters and low-quality reads; (3) mapping: align reads to the genome; (4) filtering: remove rRNAs and PCR duplicates; (5) peak calling: identify the regions enriched in IP RNA relative to input RNA. The first three are standard steps employed by all databases. However, steps (4) and (5) are executed differently by various databases as summarized in Table 2.

**Table 2:** Comparison between settings applied in databases to call m$^{6}$A

Databases	Filtering	Peak-calling	Reported-peaks	Customizable setting of parameters for peak calling?	Reported m$^{6}$A sites
MeT-DB/ MeT-DBv2.0		exomePeak	FDR<0.05, *P* <0.05, FC>1 and MAPQ>30	unavailable	Searching the RRACH motif in the identified peaks
RMBase/ RMBasev2.0		exomePeak	FDR<0.05, *P* <0.01, MAPQ>30 and FC>2	not allowed	Searching the RRACH motif in the identified peaks
CVm6A	Picard (remove PCR duplicates)	MeTPeak	FDR<0.05, FE>1 and MAPQ>30	not allowed	miCLIP-seq/PA-m$^{6}$A-seq
REPIC	Removed rRNAs, FastQ Screen	exomePeak, MeTPeak, MACS2	FDR<0.05, MAPQ>20, FE>2	not allowed	m$^{6}$A-seq and meRIP-seq
m6ASNP/ m6AVar/RMVar		MACS2, MeTPeak, Meyer’s method	Consensus peak by MSPC	not allowed	miCLIP/MAZTER-seq/m$^{6}$A-REF-seq/m$^{6}$ACE-seq/ DART-seq/PA-m$^{6}$A-Seq/m$^{6}$A-Label-seq (High confidence); meRIP-Seq (Medium confidence); Transcriptome-wide prediction (Low confidence)
m6A-Atlas/ m6A-Atlas v2.0		exomePeak2, MACS2 (v2.0), TRESS (v2.0)	Fold-enrichment>1	not allowed	m$^{6}$A-REF-seq/MAZTER-seq/miCLIP-seq/ m$^{6}$A-CLIP-seq/PA-m$^{6}$A-seq/m$^{6}$A-seq with improved protocol/m$^{6}$A-CLIP-seq
m6A-TSHub		exomePeak2	P <1e-10, contain at least one DRACH	not allowed	miCLIP/m$^{6}$A-CLIP-seq, m$^{6}$A-REF-seq and DART-seq

The levels of stringency and filtering can affect the confidence of true m$^{6}$A peaks being included in these databases. For example, some databases define RNA regions as being enriched for m$^{6}$A when peaks show fold-change (FC) > 1 against input (Table 2). However, low fold-change (FC < 1.5) of signal over input may lead to false positives. On the other hand, higher stringency of FC settings may lead to false negatives in some cell types that may have generally lower levels of m$^{6}$A compared with others. Ideally, end-users should be allowed to alter the default settings to obtain more reliable results, but this is not possible with most m$^{6}$A databases (Table 2). While some databases have included base-resolution methods to validate the presence of m$^{6}$A sites in enriched m$^{6}$A peaks, others have performed such validation based on the presence of the consensus m$^{6}$A motif, DRACH or RRACH (Table 2). It is important for end-users to recognize that not all DRACH or RRACH motifs are methylated and m$^{6}$A sites defined using such criteria require further verification.

**Figure 2:**
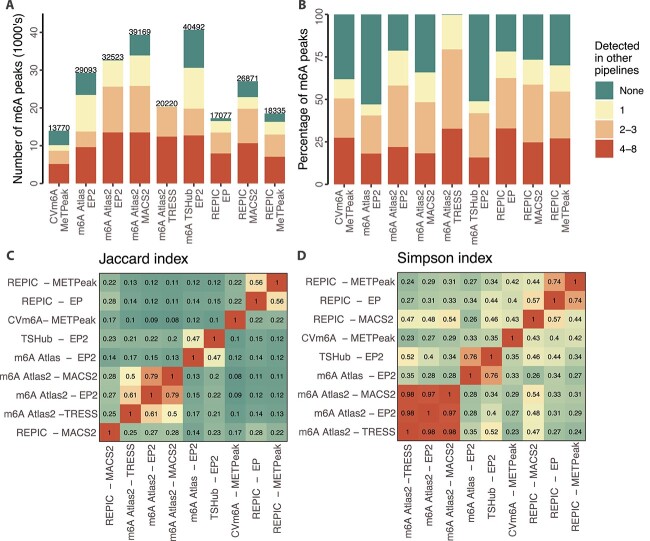
Comparison of peak calls from NOMO-1 cells (GSE87190) across nine database pipelines from five databases; (A) barplot of the total number of peaks reported by each database pipeline, with colours representing the number of other database pipelines each peak has an intersection with; colours are per the legend of (B); (B) barplot of the percentage of each database pipeline’s peak set that intersects a peak in none, one, two to three, or $\geq 4$ of the eight other database pipelines; (C, D) clustered heatmap of the Jaccard Index (C) and Simpson Index (D) for the pairwise intersections between peak coordinates reported by each database pipeline; *EP*: ExomePeak; *EP2*: ExomePeak2.

We thus compared how the same input data can produce variable output in peak calls using an exemplar m6A-seq dataset from NOMO-1 cells (GSE87190) [63]. Peak calls derived from this accession were selectable in five databases (CVm6A, m6A-Atlas, m6A-Atlas v2.0, m6A-TSHub, and REPIC), but were either absent or unavailable for download from the other databases described in this review. A total of nine different processing pipelines were compared, as two databases reported results from multiple peak calling algorithms (m6A-Atlas v2.0 and REPIC). Database pipeline stringency varied greatly, with the number of peaks ranging from 13 770 in CVm6A to approximately 40 000 in m6A-Atlas v2.0 (when using the MACS2 caller) and m6A-TSHub, nearly a three-fold difference (Fig. 2A). CVm6a may have called the fewest peaks as it filtered out PCR duplicate reads using PICARD. Indeed, within each of the nine pipelines, the percent of peak calls that failed to intersect any of the other pipelines’ averaged 30.5%, but was highly variable (range: 0.8%–53.4%) (Fig. 2B). In contrast, only an average of 24.2% of peak calls per pipeline were highly reproducible, i.e. intersected with a peak from at least half the other pipelines (Fig. 2B). The database with the fewest unique peak calls was TRESS output from m6A-Atlas v2.0 (0.8%), while ExomePeak2 output from REPIC had the highest percentage of its peaks being highly reproducible (32.4%) (Fig. 2B).

We next evaluated the pairwise concordance between each of the database pipeline peak calls. Using the Jaccard Index (number of intersecting peaks / number of peaks in set union), we observed very low similarity overall, with similarity above 0.5 only observed within databases when multiple peak callers were used (Fig. 2C). However, as Jaccard penalizes set size imbalances, we also controlled for this using Simpson Similarity (number of intersecting peaks / number of peaks in smaller set), which further highlighted that the strongest concordance was within databases rather than across them (Fig. 2C). Interestingly, the highest across-database similarity was between m6A-TSHub and m6A-Atlas, where both used ExomePeak2 as their peak caller, suggesting that algorithm choice also drives similarity. These numbers are also broadly consistent with REPIC’s statistics comparing its three peak callers, where REPIC MACS2 calls rarely reached a Jaccard similarity of >0.5 to REPIC exomePeak or MeTPeak calls within the same accession (13.6% and 3%, respectively).

We also repeated these analyses using a second accession (H1299 meRIP-seq from GSE76367 [64]), which broadly replicated these trends (Fig. S1).

Overall, we found that a single accession can produce vastly different peak calls depending on the database a user queries, owing to different (re)processing pipelines. This variability is likely in addition to that present across experiments even when controlling for the pipeline; while replicates reach approximately 80% peak overlap, this figure falls to 45% when considered across studies in a single tissue [55]. Newer databases like REPIC and m6A-Atlas v2.0 have begun to incorporate peak calls from multiple algorithms, and while this added transparency may help users prioritize reproducible results, our results show that peak calls cluster most strongly according to databases rather than algorithms, and thus that robustness should be evaluated across rather than within databases.

We note that the above example demonstrates substantial cross-database variability in the simplest case, where, for a given cell type, there exists only one accession with no replicates. However, for more common model cell types, multiple different accessions may be available, each of which may have several replicates and/or perturbations. Using HepG2 as an example, CVm6A has re-processed one accession (GSE37005), REPIC uses two (GSE37002, GSE37003), m6A-TSHub takes three (GSE90642, GSE102336, and GSE110320), and m6A-Atlas calls from four (GSE37002, GSE90642, GSE102336, and GSE110320). Given that peaks called across m6A-seq experiments already show poor concordance [55], this may serve as an additional factor reducing agreement across databases, one which end-users should be cognizant of.

### Differences in the sensitivity of the early m$^{6}$A-seq protocol and the refined RIP-seq protocol impact the accuracy of m$^{6}$A calling in databases

m$^{6}$A-seq protocol was originally reported in 2012 and has been widely used to profile m$^{6}$A in diverse cells and tissues [2]. However, to circumvent the requirement of over 300 $\mu $g of total RNA [29] or 5 $\mu $g of mRNAs, the RIP-seq protocol [27] was developed and requires as low as 0.5 $\mu $g of total RNA. For the purpose of this discussion, we termed the original m$^{6}$A-seq protocol the ‘early protocol’ (EP) and RIP-seq as the ‘refined protocol’ (RP). Most m$^{6}$A databases only collected the m$^{6}$A-seq data generated using EP [40–43, 45] while few others included data generated using RP [44, 46, 51]. Given that the sensitivity of m$^{6}$A detection using EP and RP varies considerably, the sensitivity of m$^{6}$A calling varies between databases.

**Figure 3:**
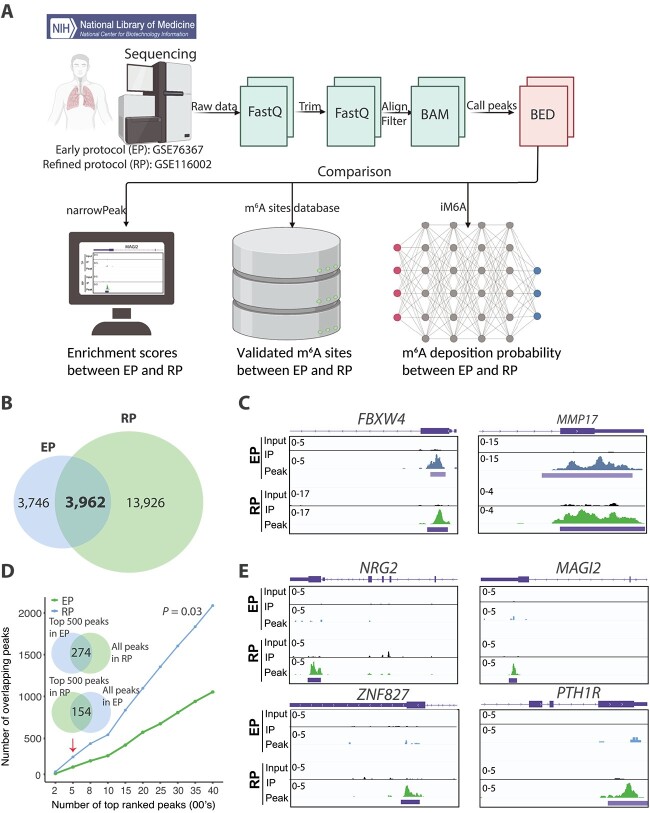
Comparison of the m$^{6}$A peaks in A549 identified using early and refined m$^{6}$A-seq protocols; (A) a workflow to process and compare data generated using the early and refined m$^{6}$A-profiling protocols; m$^{6}$A profiling data sets used in the work were generated from early m$^{6}$A-seq protocol (EP) [29] and refined RIP-seq protocol (RP) [27] for the human A549 cell line; MACS2 was used to call narrow peaks; iM6A was implemented to calculate the methylation probability of m$^{6}$A peaks (iM6A score); (B) overlap of m$^{6}$A peaks detected in the early m$^{6}$A-seq protocol (EP) and the refined RIP-seq protocol (RP); (C) IGV plots of *FBXW4* and *MMP19* showing m$^{6}$A peaks that are detectable by both EP and RP; (D) the number of overlapping m$^{6}$A peaks called by both methods in the top 4000 peaks ranked by the peaks*′* fold-enrichment; Venn diagram showing top 500 m$^{6}$A peaks detected in EP that overlap with all peaks in RP and vice versa; (E) m$^{6}$A peak signals in *NRG2*, *MAGI2*, *ZNF827* and *PTH1R* transcripts detected using RP but not EP. Parts of the figure were generated using Bio-Render.

We compared the m$^{6}$A profile of a human lung cancer cell line, A549, previously generated using EP and RP protocols, and illustrated the erroneous m$^{6}$A peak calling in databases. Both EP and RP protocols utilized single-end sequencing and immunoprecipitation were performed using the same m$^{6}$A-antibody (Table 3). The read length and sequencing depth of the different protocols were similar, thereby minimizing any biases in the quality of data analysed (Table 3) [27, 29]. We applied our pipeline in Fig. 3A to reanalyse m$^{6}$A data generated from EP and RP, and used MACS2 to call narrow peaks. We selected peaks with fold enrichment > 4 over input control, consistent with the recommended calling of reliable peaks in chromatin immunoprecipitation sequencing (ChIP-seq) data [74].

**Table 3:** Comparison of the sequencing parameters, output, and antibodies used in two studies that profiled m$^{6}$A in A549 using the early (EP) and refined (RP) protocols

Sample	SRA	RL$^{1}$$($bp$)$	RD$^{2}$$($m$^{3}$$)$	Platform	CL$^{5}$	Genome	Antibody
m$^{6}$a-seq (EP) INP	SRX1503161	50	30.9	HiSeq2000 (S$^{4}$)	A549	Human	
m$^{6}$a-seq (EP) IP	SRX1503162	50	29.9	HiSeq2000 (S$^{4}$)	A549	Human	Synaptic Systems, 202003
RIP-seq (RP) INP	SRX4239819	51	42.9	HiSeq2500 (S$^{4}$)	A549	Human	
RIP-seq (RP) IP	SRX4239820	51	33.8	HiSeq2500 (S$^{4}$)	A549	Human	Synaptic Systems, 202003

$^{1}$ Read Length; $^{2}$Read depth; $^{3}$million; $^{4}$Single-end; $^{5}$Cell Line

First, we checked the percentage overlap of peaks detected by the two protocols (Fig. 3B). In A549, the RP protocol detected three times more m$^{6}$A peaks (17 888) than those detected using EP (7708). 3962 m$^{6}$A peaks that account for 20$\%$ of peaks detected using RP overlapped with 50$\%$ of peaks detected using EP (Fig. 3B). Among the overlapping genes detected by EP and RP, we observed key oncogenes and tumour suppressor genes including *FBXW4*, *MMP17*, *RBM15*, *SETDB1*, *PAX8*, *IRS1*, *ABL2*, *HDAC4*,* MST1R*, *GATA2*, *FOXL2*, *SOX2*, *TET3*, *TMEM127*, *VHL*, *XPC*, *TRAF5*, *RASA1*, *SPRTN*, and *CDKN2C* (Figs 3C and S2), indicating the consistency in detecting m$^{6}$A genes relevant to a cancer cell line. Notably, nearly 78$\%$ of peaks detected in RP are not detected using EP, indicating that RP is more sensitive than EP (Fig. 3B).

Second, we ranked the peak signals from high to low and determined whether the top 500 peaks detected in EP are also detectable using RP. We then performed the opposite by overlapping the top 500 peaks in RP with all peaks detected using EP. RP m$^{6}$A peaks that overlapped with EP (274/500) were two times more than top EP peaks that were detectable using RP (154/500) (Fig. 3D), reaching a statistical significance of *P* < 0.03 (Kolmogorov–Smirnov test). Our analysis further suggests that peak signals detected using EP may be weaker and potentially lead to false negatives. Examples of m$^{6}$A peaks in cancer-associated genes (*NGR2*, *MAGI2*, *ZNF827*, and *PTH1R*) [75–78] detected using RP but not EP are shown in Fig. 3E. Our analysis indicates that the contribution of m$^{6}$A to these functionally essential genes may be missed if the profiling is based solely on EP.

**Figure 4:**
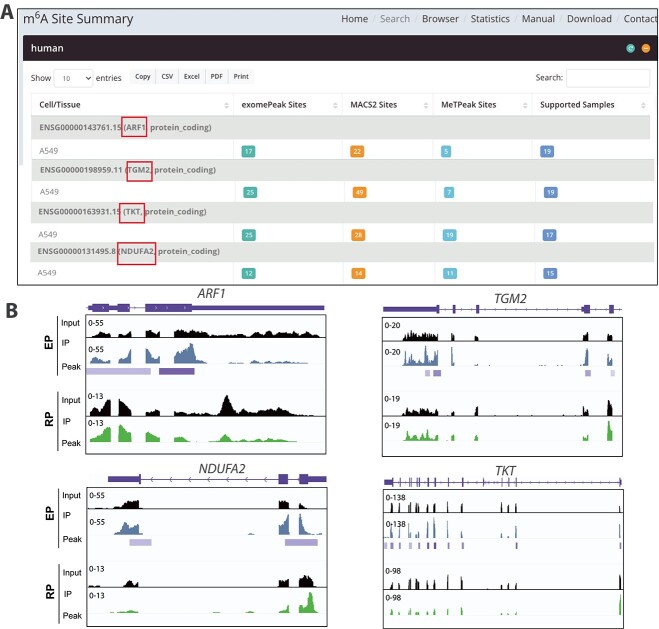
Inconsistencies of the m$^{6}$A peaks in A549 identified using early and refined m$^{6}$A-seq protocols; (A) a screenshot from REPIC that indicates m$^{6}$A sites in *ARF1*, *TGM2*, *NDUFA2,* and *TKT* in human A549 cell lines; (B) IGV plots showing m$^{6}$A peaks in *ARF1*, *TGM2*, *NDUFA2*, and *TKT* detected in EP- but not RP-generated data.

In addition, some of the peaks detected exclusively by EP need to be interpreted with caution because the m$^{6}$A profile indicates enriched peaks that mapped perfectly to all exons end-to-end, similar to the mRNA-seq profile. Illustrations of these types of peaks (generated using the EP protocol and documented in the REPIC database) are shown in Fig. 4A and 4B. The peaks (indicated by purple bars) were not identified as being enriched over input in the RP data (Fig. 4B). Thus, data generated by EP require additional filters to remove these potential false-positive peaks, which may be caused by the non-specific binding of the m$^{6}$A antibody during library preparation.

**Figure 5:**
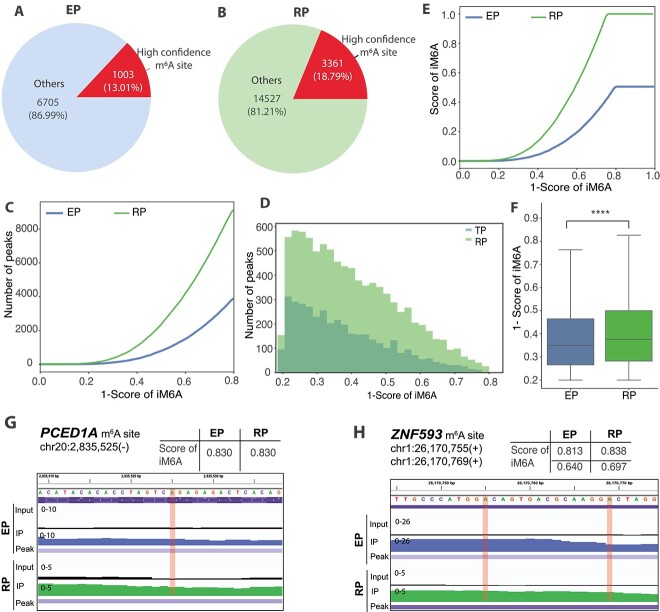
High confidence m$^{6}$A peaks in A549 called in data generated using early and refined m$^{6}$A-seq protocols via miCLIP-seq validation and iM6A scores; (A, B): the distribution of peaks containing a high confidence m$^{6}$A site detected using the early m$^{6}$A-seq protocol, EP (A), and the refined RIP-seq protocol, RP (B); (C) the distribution of m$^{6}$A scores for all identified sites; (D) histogram showing the number of m$^{6}$A sites in EP and RP, plotted against m$^{6}$A score; (E) the distribution of m$^{6}$A scores for all identified top 1000 sites (cut-off at 0.8 for x-axis); (F) boxplot of m$^{6}$A scores for top 1000 sites identified using EP and RP; (G, H): IGV plot of *PCED1A* (G) and *ZNF593*; (H) containing a peak with a high score of iM6A in data generated using EP and RP; m$^{6}$A sites with a high iM6A score are highlighted in orange; the table above the plots shows iM6A scores.

Third, we checked the overlap of the high confidence m$^{6}$A sites in the human A549 cell line among all detected m$^{6}$A sites (Fig. 5A and 5B). As shown in Fig. 5B, m$^{6}$A peaks detected by the RP protocol reported a higher overlap percentage (18.79$\%$, 3361) than the EP protocol (13.01$\%$, 1003) in Fig. 5A. Furthermore, we ranked the m$^{6}$A peaks detected via EP and RP protocols separately using the scores generated by a recently published predictor of m$^{6}$A sites based on deep-learning (iM6A) [73]. We found that for sites with high confidence iM6A scores (probability value > 0.1), RP detected twice as many m$^{6}$A sites as EP (Fig. 5C and 5D). We further examined 1000 m$^{6}$A sites with top-ranked iM6A scores and found that m$^{6}$A sites generated using RP have significantly higher iM6A scores with a *P* value of 1e-230 (Fig. 5E and 5F). While in some cases both EP and RP protocols can discover peaks with equally high iM6A scores (Fig. 5G and 5H, Fig. S3), in many cases, the iM6A score imputed by the RP method was higher than that of EP. This result indicates that the RP method of m$^{6}$A profiling is typically more sensitive. Collectively, understanding the protocols used to generate the m$^{6}$A data is essential to determine whether m$^{6}$A are likely to be correctly identified in mRNA transcripts presented in m$^{6}$A-related databases.

### The association between m$^{6}$A and SNPs reported in databases

**Figure 6:**
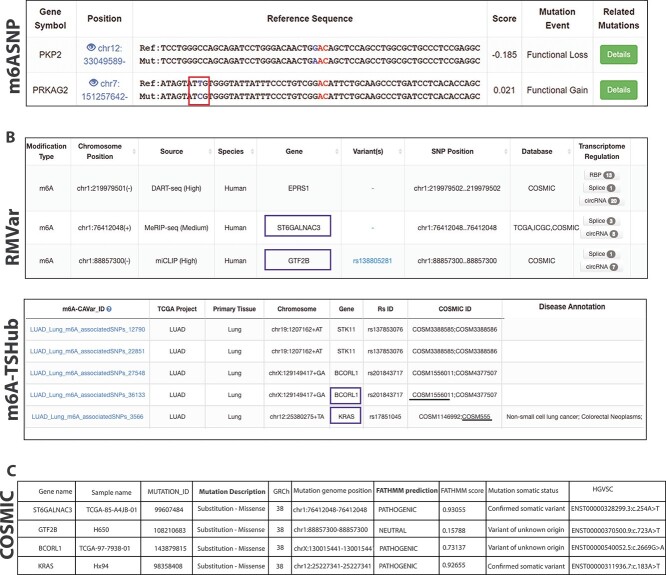
Issues of m$^{6}$A-related SNP databases; (A) screenshot showing examples of SNPs outside m$^{6}$A motifs; red rectangles are used to highlight the SNP variant; (B) screenshot from RMVar and m6A-TSHub showing high-confidence mutations that altered m$^{6}$A motifs; selected SNPs are underlined; blue rectangles are used to highlight the example of genes; (C) mutation information from COSMIC for cases highlighted in blue rectangles in (B).

The presence of SNPs within the DRACH motifs can lead to the loss or gain of m$^{6}$A sites, thereby affecting the expression, stability, and translation of mRNAs. Most databases employed a random forest model to predict whether the methylation status of a m$^{6}$A site is altered by variants that are within the flanking regions 30 nucleotides (both upstream and downstream) of a given m$^{6}$A residue in the DRACH motif. One example is shown in Fig. 6A where *PPKAG2* has a SNP (highlighted in red rectangle) which is 21bp apart from the DRACH motif.

In addition, databases do not include information on the status of SNPs within m$^{6}$A sites regarding whether they are synonymous or non-synonymous SNPs. If an SNP within a given m$^{6}$A site is non-synonymous and located in a coding exon, the SNP will lead to amino acid substitution. Consequently, the pathogenicity of the SNP cannot be attributed to a change in m$^{6}$A alone. Four examples to illustrate this scenario extracted from RMVar and m6A-TSHub are shown in blue rectangles in Fig. 6B and 6C. According to the Catalogue of Somatic Mutations in Cancer (COSMIC) database, all these four high-confidence somatic variants are described as missense and at least three of them may lead to disease pathogenicity regardless of whether m$^{6}$A is present at these sites (Fig. 6C).

### Towards overcoming the pitfalls in m$^{6}$A-related databases generated based on peak-calling experiments

Several improvements can be performed to generate reliable m$^{6}$A-related databases that will benefit non-specialist end-users. First, m$^{6}$A-related databases that incorporate data from peak-calling experiments should disclose information regarding the m$^{6}$A-profiling methods used to obtain the data and allow end-users to select data that they desire based on analysis methods. Second, end-users should be allowed to alter the stringency of the analysis method used to minimize false positive and negative results. Third, datasets included in databases should be reanalysed using superior tools such as TRESS rather than MACS2 or exomePeak to correct for biological variance between replicates and improve the accuracy [79]. Indeed, the analysis using TRESS in m6A-Atlas v2.0 revealed fewer but more reproducible peaks compared with other methods, which indicates that additional peaks detected by other methods may not be as robust. Where available, METTL3 depletion experiments performed in parallel should be included as negative controls to identify true m$^{6}$A peaks in databases. Fourth, the iM6A model can be incorporated into databases to predict the probability of m$^{6}$A peaks being real. This feature was not previously possible in most m$^{6}$A-related databases as they were created prior to the publication of iM6A. Finally, databases reporting m$^{6}$A-associated SNPs should contain a filter for synonymous and non-synonymous SNPs and focus on SNPs in the DRACH motifs that are confirmed to be methylated.

## Conclusion and future work

In conclusion, while m$^{6}$A databases provide a rich source to infer biological roles of m$^{6}$A in different cellular contexts, the understanding of how the data were generated and parameters that have been set to call m$^{6}$A are critical to derive biologically meaningful conclusions. By discussing the variability and undisclosed information related to m$^{6}$A databases we hope that end-users will become aware of ‘traps’ that may lead to inaccurate interpretation of data. Some of these ‘traps’ arise consequent to issues associated with older m$^{6}$A-seq technologies such as non-specific binding of antibodies and arbitrary cut-off used to call m$^{6}$A, and not due to variability in the data processing parameters per se. Recently, long-read techniques, such as Oxford nanopore sequencing, provided a novel way to measure m$^{6}$A levels for each adenosine at a transcriptomic scale, but few large-scale studies are available due to higher sequencing costs to acquire sufficient sequencing depth [80]. Similarly, base-resolution m$^{6}$A profiling methods analogous to bisulfite-sequencing of DNA methylation have recently been developed including GLORI [61], m$^{6}$A-SAC-seq [81], and eTAM-seq [62] but very few studies are currently available. Future generation of databases based on these base-resolution methods of m$^{6}$A profiling may provide more reliable resources to mine m$^{6}$A datasets with higher accuracy. However, until that becomes possible, databases generated based on antibody-based techniques are likely to be utilized and continue to be created. Our recommendations should give future creators of m$^{6}$A-related databases insights into creating more useful databases that will benefit non-specialist end-users. As antibody-based methods have also been used to profile other mRNA modifications including m$^{1}$A, m$^{6}$Am, and 5hmC, lessons learned from m$^{6}$A-related databases would be applicable to interpreting the data in current and future databases created for these emerging RNA modifications.

**Key Points**

- Current m$^{6}$A databases re-call peaks from sequencing data. Differences in processing pipelines drive variability in peak number and coordinates, with only moderate reproducibility.
- Databases that report m$^{6}$A peaks do not explicitly disclose the protocols used for generating the m$^{6}$A-immunoprecipitation sequencing data for each sample. We reveal that a more recent refined RIP-seq protocol is more sensitive and reliable than the m$^{6}$A-seq protocol developed prior. The inclusion of data generated using the early m$^{6}$A-seq protocol needs further filtering to avoid false positives.
- Several m$^{6}$A-related databases reported m$^{6}$A-associated SNPs that are located over 20 nucleotides from m$^{6}$A sites, raising the question of whether they lead to altered m$^{6}$A. Moreover, databases do not include information related to synonymous and non-synonymous SNPs. Non-synomymous SNPs may lead to functional consequences independently of m$^{6}$A status.

## Supplementary Material

SupplementaryInformation_bbae434

## Data Availability

Raw data files for the two independent datasets used to compare the m$^{6}$A profile of the human lung cancer cell line, A549, previously generated using EP and RP protocols are freely available for download via their GEO accession number GSE76367 and GSE116002, respectively. For NOMO-1 and H1299 peak calls accessed from databases, links can be found in Supplementary Tables S1 and S2, respectively.
