# Supplementary material for: Variable calling of m6A and associated features in databases: a guide for end-users
Source: Brief Bioinform. 2024 Sep 11;25(5):bbae434. doi: 10.1093/bib/bbae434 (PMC11388104; doi:10.1093/bib/bbae434)
Supplement: SupplementaryInformation_bbae434 [file supplementaryinformation_bbae434.docx]

**Figure S1.** Comparison of peak calls from H1299 cells (GSE76367) across nine database pipelines from five databases. (A) Barplot of the total number of peaks reported by each database pipeline, with colours representing the number of other database pipelines each peak has an intersection with. Colours are per the legend of (B). (B) Barplot of the percentage of each database pipeline’s peak set that intersect a peak in none, one, two to three, or ≥ 4 of the eight other database pipelines. (C, D) Clustered heatmap of the Jaccard Index (C) and Simpson Index (D) for the pairwise intersections between peak coordinates reported by each database pipeline. *EP*: ExomePeak. *EP2*: ExomePeak2.

**Figure S2.** IGV plots of key oncogenes and tumour suppressor genes in diverse cancers showing m^6^A peaks that are detectable by both EP and RP. *RBM15*, *SETDB1*, *HDAC4*, *JARID2* and *FOXL2* can function as either a tumour suppressor or oncogene in distinct cellular contexts. *JUN*, *SOX2*, *GATA2* and *IRS1* are known oncogenes. *TET3*, *TMEM127* and *CDKN2C* are known to be tumour suppressor genes.


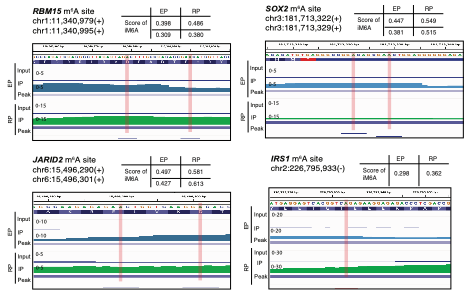


**Figure S3.** IGV plot of four key oncogenes and tumour suppressor genes containing a peak with a high score of iM6A in data generated using EP and RP.

**Table S1.** Database download links for NOMO-1 peak calls (GSE87190).

Database Pipeline URL

CVm6A / MetPeak http://gb.whu.edu.cn:8080/storage/cdm6a.tar.gz

m6A-Atlas / ExomePeak2 <http://180.208.58.19/m6A-Atlas/Download/human_peakCalling/GSE87190.zip>

m6A-Atlas v2.0 / ExomePeak2 [http://www.rnamd.org/m6a/repository_low_download.php?sp=Human&region=&seq=All&pos_start=&pos_end=](http://www.rnamd.org/m6a/repository_low_download.php?sp=Human&region&seq=All&pos_start&pos_end) [call_peak_methods=exomePeak2&condition=GSE87190;NOMO-1;Ctrl](call_peak_methods%3DexomePeak2%26condition%3DGSE87190%3BNOMO-1%3BCtrl)

m6A-Atlas v2.0 / TRESS [http://www.rnamd.org/m6a/repository_low_download.php?sp=Human&region=&seq=All&pos_start=&pos_end=](http://www.rnamd.org/m6a/repository_low_download.php?sp=Human&region&seq=All&pos_start&pos_end) [call_peak_methods=TRESS&condition=GSE87190;NOMO-1;Ctrl](call_peak_methods%3DTRESS%26condition%3DGSE87190%3BNOMO-1%3BCtrl)

m6A-Atlas v2.0 / MACS2 [http://www.rnamd.org/m6a/repository_low_download.php?sp=Human&region=&seq=All&pos_start=&pos_end=](http://www.rnamd.org/m6a/repository_low_download.php?sp=Human&region&seq=All&pos_start&pos_end) [call_peak_methods=MACS2&condition=GSE87190;NOMO-1;Ctrl](call_peak_methods%3DMACS2%26condition%3DGSE87190%3BNOMO-1%3BCtrl)

REPIC / MACS2 [https://repicmod.uchicago.edu/repic/data/download/m6A=sites=cell=human=hg38=NOMO-1.txt.gz](https://repicmod.uchicago.edu/repic/data/download/m6A%3Dsites%3Dcell%3Dhuman%3Dhg38%3DNOMO-1.txt.gz) REPIC / ExomePeak [https://repicmod.uchicago.edu/repic/data/download/m6A=sites=cell=human=hg38=NOMO-1.txt.gz](https://repicmod.uchicago.edu/repic/data/download/m6A%3Dsites%3Dcell%3Dhuman%3Dhg38%3DNOMO-1.txt.gz) REPIC / MetPeak [https://repicmod.uchicago.edu/repic/data/download/m6A=sites=cell=human=hg38=NOMO-1.txt.gz](https://repicmod.uchicago.edu/repic/data/download/m6A%3Dsites%3Dcell%3Dhuman%3Dhg38%3DNOMO-1.txt.gz) m6A-TSHub <http://180.208.58.19/tshub/download/NOMO-1.csv>

**Table S2.** Database download links for H1299 peak calls (GSE76367).

Database Pipeline URL

CVm6A / MetPeak http://gb.whu.edu.cn:8080/storage/cdm6a.tar.gz

m6A-Atlas / ExomePeak2 <http://180.208.58.19/m6A-Atlas/Download/human_peakCalling/GSE76367.zip>

m6A-Atlas v2.0 / ExomePeak2 [http://www.rnamd.org/m6a/repository_low_download.php?sp=Human&region=&seq=All&pos_start=&pos_end=](http://www.rnamd.org/m6a/repository_low_download.php?sp=Human&region&seq=All&pos_start&pos_end) [call_peak_methods=exomePeak2&condition=GSE87190;H1299;Ctrl](call_peak_methods%3DexomePeak2%26condition%3DGSE87190%3BH1299%3BCtrl)

m6A-Atlas v2.0 / TRESS [http://www.rnamd.org/m6a/repository_low_download.php?sp=Human&region=&seq=All&pos_start=&pos_end=](http://www.rnamd.org/m6a/repository_low_download.php?sp=Human&region&seq=All&pos_start&pos_end) [call_peak_methods=TRESS&condition=GSE87190;H1299;Ctrl](call_peak_methods%3DTRESS%26condition%3DGSE87190%3BH1299%3BCtrl)

m6A-Atlas v2.0 / MACS2 [http://www.rnamd.org/m6a/repository_low_download.php?sp=Human&region=&seq=All&pos_start=&pos_end=](http://www.rnamd.org/m6a/repository_low_download.php?sp=Human&region&seq=All&pos_start&pos_end) [call_peak_methods=MACS2&condition=GSE87190;H1299;Ctrl](call_peak_methods%3DMACS2%26condition%3DGSE87190%3BH1299%3BCtrl)

REPIC / MACS2 [https://repicmod.uchicago.edu/repic/data/download/m6A=sites=cell=human=hg38=H1299.txt.gz](https://repicmod.uchicago.edu/repic/data/download/m6A%3Dsites%3Dcell%3Dhuman%3Dhg38%3DH1299.txt.gz) REPIC / ExomePeak [https://repicmod.uchicago.edu/repic/data/download/m6A=sites=cell=human=hg38=H1299.txt.gz](https://repicmod.uchicago.edu/repic/data/download/m6A%3Dsites%3Dcell%3Dhuman%3Dhg38%3DH1299.txt.gz) REPIC / MetPeak [https://repicmod.uchicago.edu/repic/data/download/m6A=sites=cell=human=hg38=H1299.txt.gz](https://repicmod.uchicago.edu/repic/data/download/m6A%3Dsites%3Dcell%3Dhuman%3Dhg38%3DH1299.txt.gz) m6A-TSHub <http://180.208.58.19/tshub/download/H1299.csv>
